# Modified enhanced pleural fixation: combined intra- and postoperative hypertonic glucose in elderly and high-risk pulmonary bullae patients — a retrospective cohort study

**DOI:** 10.1186/s12893-026-03826-y

**Published:** 2026-05-21

**Authors:** Wenlong Chen, Bingxia Yang, Zhuang Zuo, Changhao Que, Yaling Liu, Qi Wang, Ying Ma, Songchen Han, Yunjiu Gou

**Affiliations:** 1 The First Clinical College of Gansu University of Traditional Chinese Medicine, Lanzhou, 730000 China; 2 Department of Thoracic Surgery, Gansu Provincial People’s Hospital, Lanzhou, 730000 China

**Keywords:** Pleural fixation surgery, Hypertonic glucose, Thoracoscopic surgery, Pulmonary bullae, Spontaneous pneumothorax

## Abstract

**Background:**

Although mechanical pleurectomy effectively prevents recurrent pneumothorax in elderly patients with diffuse emphysematous bullae, it entails substantial surgical trauma and postoperative pain. This study evaluates an enhanced pleurodesis technique—combining intraoperative hypertonic glucose spraying with three-day postoperative intrapleural perfusion—as a minimally invasive alternative.

**Methods:**

A retrospective analysis included 155 patients who underwent thoracoscopic bullectomy: 72 in the glucose protocol group and 83 in the parietal pleurectomy control group. Following 1:1 propensity score matching, 72 well-balanced matched pairs were obtained.

**Results:**

The glucose protocol group exhibited significantly improved perioperative outcomes compared with the parietal pleurectomy control group, including shorter operative duration (65.33 ± 2.43 vs. 84.92 ± 2.35 min), reduced intraoperative blood loss (50.00 ± 3.82 vs. 71.89 ± 4.62 mL), lower postoperative chest tube drainage volume (551.39 ± 27.76 vs. 785.42 ± 57.83 mL), shorter chest tube dwell time (4.42 ± 0.17 vs. 5.32 ± 0.21 days), lower pain scores on postoperative day 3 (0.93 ± 0.07 vs. 1.28 ± 0.06), and shorter length of hospital stay (5.04 ± 0.16 vs. 6.31 ± 0.24 days) (all *P* < 0.05). No significant differences were observed in postoperative complication rates (18.1% vs. 15.3%) or one-year pneumothorax recurrence rates (4.2% vs. 2.8%) between the two groups (both *P* > 0.05).

**Conclusion:**

In this retrospective, propensity-score matched cohort study involving elderly high-risk patients with recurrent diffuse pulmonary bullae, an enhanced pleurodesis protocol—comprising intraoperative hypertonic glucose spray followed by three-day postoperative intrapleural perfusion—was associated with significantly improved perioperative outcomes compared with parietal pleurectomy. No statistically significant difference was detected in 1-year recurrence; however, this finding should not be interpreted as evidence of equivalence or non-inferiority because the recurrence analysis was underpowered and no non-inferiority margin was prespecified. These results indicate that the glucose-based protocol may serve as a viable, less invasive alternative to mechanical pleurectomy in this vulnerable population; however, validation through prospective, randomized controlled trials remains essential to establish causality and generalizability.

**Supplementary Information:**

The online version contains supplementary material available at 10.1186/s12893-026-03826-y.

## Introduction

Spontaneous pneumothorax is an acute respiratory condition primarily caused by rupture of pulmonary bullae, resulting in partial or complete lung collapse and presenting with symptoms such as pleuritic chest pain and dyspnea [1].With global population aging, its incidence among elderly patients is rising. Older adults frequently have comorbid pulmonary disorders—including emphysema and chronic obstructive pulmonary disease (COPD)—which predispose them to atypical clinical presentations, increased diagnostic uncertainty, and higher one-year recurrence rates (25%–30%) [2–5].Recurrent episodes markedly impair health-related quality of life and impose a substantial economic and resource burden on healthcare systems, highlighting the critical need for effective pleural symphysis—achieved through minimally invasive or conventional pleurodesis techniques—to prevent recurrence [6].

Conventional pleural fixation strategies encompass mechanical and chemical pleurodesis. Mechanical pleurectomy—typically involving parietal pleural abrasion or stripping—induces pleural symphysis through controlled injury to the pleural surface; however, it is associated with substantial intraoperative bleeding, pronounced postoperative pain, and prolonged operative time [7, 8].Its efficacy may be further compromised in patients with impaired wound healing capacity, such as those with advanced age, malnutrition, or comorbid COPD [9].In contrast, chemical pleurodesis employs sclerosing agents—including sterile talc slurry and hypertonic glucose solution—to provoke a localized inflammatory response that promotes fibrous adhesion formation. This approach is technically less demanding and better tolerated, yet historically linked to higher rates of pneumothorax recurrence and, rarely, systemic inflammatory reactions [10, 11].

In elderly and high-risk patients with recurrent, diffuse, or multiple pulmonary bullae refractory to conservative management, thoracoscopic bullectomy alone is insufficient to prevent pneumothorax recurrence; adjunctive pleural symphysis is therefore essential. In patients with secondary spontaneous pneumothorax secondary to diffuse emphysematous bullae, bullectomy alone is associated with unacceptably high recurrence rates. Horio et al. reported that video-assisted thoracoscopic bullectomy without adjunctive pleurodesis resulted in a 16% 1-year recurrence rate—significantly higher than the 1.9% observed with concomitant pleurodesis [12]. A 2022 network meta-analysis confirmed that bullectomy combined with chemical pleurodesis, mechanical pleurodesis, or staple line coverage significantly reduces recurrence compared with bullectomy alone [13]. The 2024 joint ERS/EACTS/ESTS clinical practice guidelines recommend adjunctive pleurodesis at the time of surgical intervention for spontaneous pneumothorax to reduce recurrence risk [8]. Thus, the necessity of some form of pleural symphysis in this population is well established; the unresolved clinical question concerns which pleurodesis strategy offers the optimal risk–benefit profile for elderly and high-risk patients.

This study was not designed to identify a universally optimal intervention for secondary spontaneous pneumothorax; rather, it aimed to compare two adjunctive pleural symphysis strategies—intraoperative hypertonic glucose spray followed by three-day postoperative intrapleural glucose perfusion (an enhanced chemical pleurodesis protocol) versus parietal pleurectomy—in elderly high-risk patients with diffuse emphysematous bullae. The primary endpoints were perioperative morbidity, procedural safety, and the 1-year recurrence rate of pneumothorax. As no bullectomy-only control group was included, the incremental benefit and risk attributable specifically to pleurodesis—beyond those conferred by bullectomy alone—cannot be isolated or quantified. Consequently, our findings represent a direct comparative evaluation of two pleural symphysis approaches, not an ordinal ranking of interventions across the broader therapeutic spectrum.

## Methods

### Materials and methods

#### Baseline characteristics before and after PSM

This retrospective cohort study is reported in accordance with the Strengthening the Reporting of Observational Studies in Epidemiology (STROBE) guidelines. It included patients who underwent thoracoscopic bullectomy for secondary spontaneous pneumothorax at the Department of Thoracic Surgery, Gansu Provincial People’s Hospital, between January 2020 and December 2024. Of the 155 patients initially enrolled, all met predefined inclusion criteria. The choice of adjunctive pleurodesis strategy was made in routine clinical practice and was not randomized, protocol-mandated, or standardized across surgeons. Before surgery, the attending thoracic surgeon selected the pleurodesis approach after reviewing the patient’s CT findings, the extent and distribution of emphysematous bullae, anticipated technical feasibility of parietal pleurectomy, expected tolerance of pleurectomy-related bleeding and postoperative pain, comorbidity burden, and patient/family preference. Parietal pleurectomy was generally used when durable mechanical pleural symphysis was considered feasible and the patient was judged able to tolerate a more invasive pleural procedure. The glucose protocol was increasingly used when lower tissue trauma was preferred, particularly in patients with diffuse emphysematous bullae, anticipated difficult pleural dissection, limited physiological reserve, or concern for postoperative pain or bleeding. Therefore, treatment choice was surgeon-dependent and partly influenced by evolving departmental practice over the study period. These selection factors were incompletely captured in the retrospective dataset and may have contributed to residual confounding despite propensity score matching. The baseline characteristics before and after matching are presented in Table 1.

**Table 1:** Comparison of baseline characteristics before and after propensity score matching

Characteristic	Before matching			After matching		
	Observation Group *n *= 72	Control Group *n* = 83	*P*	Observation Group *n* = 72	Control Group *n* = 72	*p*
Age (years)	56.93 ± 1.26	57.10 ± 1.22	0.926	56.89 ± 1.31	56.95 ± 1.28	0.921
Gender (Male, %)	83.3	91.6	0.119	83.3	84.7	0.857
BMI(kg/m²)	20.75 ± 0.22	20.12 ± 0.27	0.079	20.73 ± 0.23	20.68 ± 0.25	0.862
Smoking history (Yes, %)	72.2	71.1	0.875	72.2	70.8	0.881
Pneumothorax location (right side, %)	59.7	48.2	0.151	59.7	61.1	0.874
Diabetes (Yes, %)	16.7	19.3	0.674	16.7	15.3	0.852
Hypertension (Yes, %)	12.5	14.5	0.728	12.5	13.9	0.836
COPD (Yes, %)	8.3	9.6	0.763	8.3	9.7	0.819
Emphysema severity (Goddard score)						
Mild (≤ 8), n (%)	33 (45.8)	36 (43.4)	0.763	33 (45.8)	32 (44.4)	0.862
Moderate (9–16),n (%)	29 (40.3)	35 (42.2)	0.802	29 (40.3)	30 (41.7)	0.859
Severe (> 16), n (%)	10 (13.9)	12 (14.5)	0.915	10 (13.9)	10 (13.9)	0.999

*Data are presented as mean ± standard deviation or *n* (%). All standardized mean differences (SMDs) after matching are < 0.1, indicating adequate baseline balance. All patients had CT-confirmed diffuse emphysematous

##### General information

This includes age, gender, body mass index (BMI), pneumothorax laterality (left or right), smoking history (defined as regular smoking for more than one year and continued smoking within one month before surgery), and comorbidities (including hypertension, diabetes, and chronic obstructive pulmonary disease; all diagnoses were confirmed based on documented preoperative medical records). For the purposes of this study, the term “elderly” was operationally defined as age ≥ 50 years. This threshold is supported by epidemiological evidence indicating that spontaneous pneumothorax exhibits a bimodal age distribution, with a secondary peak comprising patients aged ≥ 50 years, among whom approximately 90% have underlying lung disease including pulmonary emphysema [2]. Furthermore, age > 50 years has been identified as a dominant predictive factor for distinguishing secondary from primary spontaneous pneumothorax [14]. The designation “high-risk” was defined a priori based on the presence of one or more of the following objective criteria: (1) CT-confirmed diffuse emphysematous bullae with Goddard scores ≥ 9, a threshold independently associated with prolonged air leak and recurrence in secondary spontaneous pneumothorax [15, 16]; (2) documented diagnosis of COPD, which confers a significantly elevated risk for postoperative morbidity and mortality following thoracic surgery [17]; (3) age ≥ 65 years, a recognized risk factor per Chinese multidisciplinary perioperative airway management consensus guidelines; or (4) American Society of Anesthesiologists (ASA) physical status ≥ III, which has been independently validated as a strong predictor of postoperative morbidity in elderly patients undergoing thoracoscopic surgery [18]. These criteria were prospectively established for this cohort, grounded in clinical evidence and aligned with intraoperative decision-making patterns that favor minimally invasive pleurodesis approaches. The context-dependent nature of these operational definitions is explicitly acknowledged in the Limitations section.

##### Perioperative indicators

This includes surgical duration, intraoperative blood loss, total drainage volume, duration of indwelling catheter, postoperative hospital stay, and postoperative pain score. Surgical duration was defined as the time interval from initial skin incision to completion of wound closure. Postoperative intrapleural instillation of 50% hypertonic glucose solution was administered at the bedside via the indwelling chest tube and—per internationally recognized surgical outcomes reporting standards (e.g., STROBE, CONSORT-Surgery)—was explicitly excluded from surgical duration. Accordingly, all protocol-specified postoperative interventions—including the 6-hour postural drainage period and daily chest tube clamping—were classified and analyzed as components of postoperative care, not intraoperative time. Postoperative pain was evaluated using a 0–10 visual analogue scale (VAS) on postoperative days 1–3 as part of routine ward nursing assessment. Assessments were performed by trained ward nurses at standardized daily postoperative assessment points. Because of the retrospective design and the visibly different postoperative management pathways, assessors were not blinded to pleurodesis strategy. Both groups received the same institutional postoperative analgesic protocol, including patient-controlled intravenous analgesia for up to 48 h or until extubation and supplemental analgesics as clinically required.

##### Postoperative outcome indicators

This includes persistent pulmonary air leakage, pleural effusion, pulmonary infection, transient hyperglycemia, intercostal nerve injury, encapsulated pleural effusion, thoracic residual cavity, and recurrence rate.

### Inclusion and exclusion criteria

Inclusion criteria: (1) Patients with secondary spontaneous pneumothorax definitively attributed to diffuse, multiple pulmonary bullae associated with emphysema, as confirmed by high-resolution chest CT; (2) Patients experiencing recurrent pneumothorax episodes despite conservative management; (3) Patients without contraindications to general anesthesia or thoracoscopic surgery.

Exclusion criteria: (1) Intraoperative unplanned events requiring conversion to thoracotomy; (2) Presence of surgical contraindications such as severe organ dysfunction; (3) Preexisting empyema, hemothorax, or malignant pleural effusion; (4) Uncontrolled diabetes or known hypersensitivity to glucose solution.

### Emphysema severity assessment

Emphysema severity was quantified using the Goddard scoring system—a validated, semi-quantitative, CT-based method for assessing the spatial extent of emphysematous lung destruction [19, 20]. For each patient, three standardized transverse CT sections were selected: at the level of the aortic arch (upper lung zone), at the level of the carina (middle lung zone), and 1 cm above the right hemidiaphragm (lower lung zone). Within each section, the left and right lungs were evaluated independently, yielding six anatomically defined lung regions per patient. Each region was assigned a score (0–4) based on the proportion of low-attenuation areas ( < − 950 HU) consistent with emphysema: 0 = none; 1 = ≤ 25%; 2 = 26–50%; 3 = 51–75%; 4 = > 75% [21, 22]. The total Goddard score—ranging from 0 to 24—was calculated as the sum of the six regional scores. Patients were stratified into three severity categories according to this aggregate score: mild (≤ 8), moderate (9–16), and severe (> 16) [20].All scoring was performed independently by two board-certified thoracic radiologists blinded to clinical data and study outcomes. Interobserver agreement was assessed using Cohen’s kappa coefficient (κ = 0.86), indicating excellent inter-rater reliability.

### Therapeutic method

#### Surgical approach

**Fig. 1:**
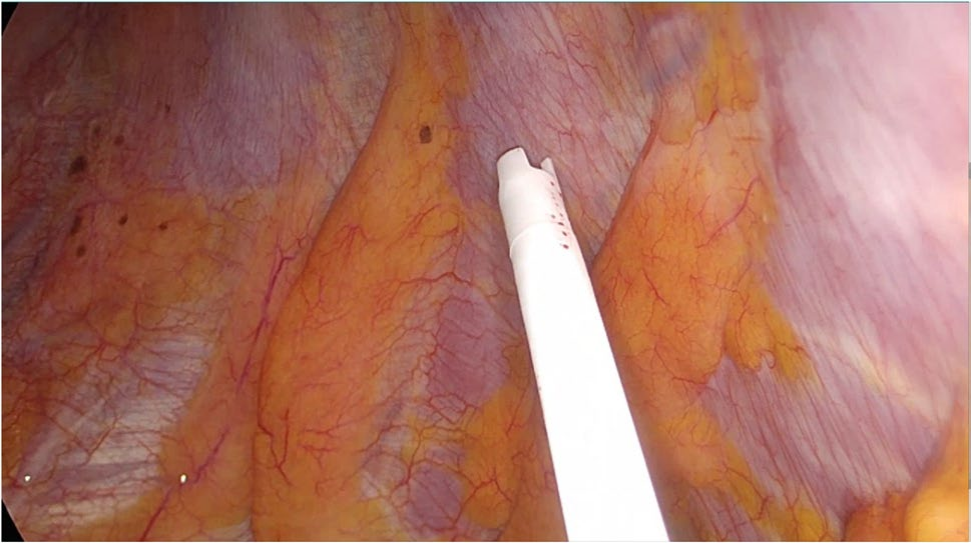
Hypertonic glucose spraying is underway intraoperatively

**Fig. 2:**
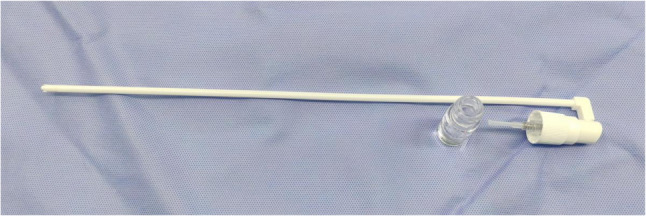
The hypertonic glucose spraying device adopts the Kangpaiter medical adhesive (1.5 endoscopic type)

All patients received general anesthesia with double-lumen endotracheal intubation and were positioned laterally on the healthy side. The surgery employs the two-port approach: An observation port of approximately 1–1.5 cm in length is created at the 7th intercostal space along the mid-axillary line, and an operative port of approximately 2–3 cm in length is established at the 4th intercostal space between the anterior axillary line and the mid-axillary line. Under thoracoscopic guidance, a linear cutting stapler is used to resect pulmonary bullae, while smaller bullae are managed with ligation or electrocautery. Following completion of resection, a water-seal test was performed to detect intraoperative pulmonary air leakage. The presence or absence of air leakage was systematically documented in the operative notes. In cases of persistent air leakage, targeted surgical intervention—including additional suturing or stapling—was undertaken until cessation of bubble formation was confirmed under water immersion. Subsequently, group-specific interventions were implemented: In the observation group, 100 mL of sterile 50% glucose solution was evenly sprayed onto the pleural surface via the operating port, with emphasis on covering the thoracic apex, costophrenic angles, and paravertebral sulci (Figs. 1 and 2).

All procedures were performed by attending thoracic surgeons within the same department using a standardized two-port VATS workflow; however, procedures were not necessarily performed by the same individual surgeon. Differences in surgeon experience, intraoperative judgment, and technical preference may have influenced both treatment selection and perioperative outcomes. Moreover, parietal pleurectomy is intrinsically more technically demanding than topical hypertonic glucose spraying because it requires pleural incision, dissection, and hemostasis over a broad parietal pleural surface.

**Fig. 3:**
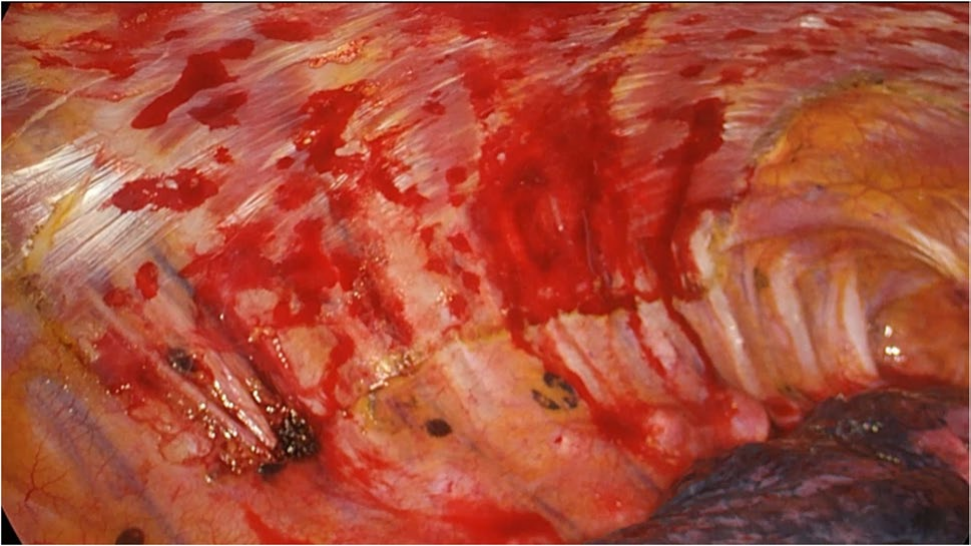
The pleura after parietal pleurectomy

In the control group, an electrocoagulation hook was used to make incisions within the predetermined range: extending cranially to the 3rd rib, anteriorly to a point 3 cm lateral to the internal thoracic and pulmonary arteries, posteriorly to a point 3 cm lateral to the sympathetic nerve, and inferiorly to the 8th rib, to incise the parietal pleura. After clamping the pleural edge with a double-jaw forceps, rotational dissection was performed with careful preservation of the sympathetic nerve. Finally, hemostasis was achieved and the surgical site was cleaned (Fig. 3).

Both groups received routine postoperative management, including drainage tube placement, wound suturing, and supportive treatments such as anti-infective therapy, analgesia, and nutritional support.

#### Postoperative management and follow-up

Following surgical intervention, all patients underwent chest tube placement. In the glucose protocol group, the following standardized postoperative management strategy was implemented: (1) Immediate postoperative positioning: Upon completion of thoracoscopic bullectomy and intraoperative instillation of 50% hypertonic glucose solution, air leakage was confirmed to be negligible via underwater seal bubble testing. Subsequently, the chest tube was clamped, and patients were allowed to awaken from general anesthesia in the operating room. Endotracheal extubation was performed prior to transfer to either the post-anesthesia care unit (PACU) or the surgical ward for a structured 6-hour postural drainage period. During this interval, patients—while fully awake and cooperative—underwent systematic repositioning (supine → left lateral → right lateral → prone) at approximately 30-minute intervals to ensure homogeneous distribution of the sclerosing agent across the pleural surface. Continuous monitoring was maintained throughout for signs of respiratory distress, including dyspnea, oxygen desaturation (SpO₂ < 90% on room air), and subcutaneous emphysema. No adverse events attributable to the protocol occurred during any repositioning cycle. (2) Postoperative intrapleural glucose perfusion: On postoperative days 1–3, 20 mL of 50% dextrose solution was instilled through the chest tube, followed by a 2-hour clamping period. Clamping was initiated only upon fulfillment of two objective criteria: (i) absence of active air leakage—defined as no continuous bubbling in the underwater seal drainage system—and (ii) complete lung re-expansion, confirmed by upright posteroanterior chest radiography. During each clamping interval, patients were continuously monitored for dyspnea, oxygen desaturation (SpO₂ < 90% on room air), and subcutaneous emphysema. No protocol-related adverse events—including tension pneumothorax, severe respiratory distress, or clinically significant subcutaneous emphysema—occurred during any clamping period. Chest tube removal was performed when all of the following criteria were met: (i) 24-hour pleural drainage volume < 100 mL; (ii) absence of air leakage; (iii) clear breath sounds on auscultation; and (iv) complete lung re-expansion confirmed by upright posteroanterior chest radiography. Analgesia was administered via a patient-controlled intravenous analgesia (PCIA) pump for up to 48 h or until extubation, with supplemental intravenous or oral analgesics titrated to patient need. Discharge eligibility required resolution of fever, clear breath sounds on auscultation, and radiographic confirmation of complete lung re-expansion without residual air or fluid on upright posteroanterior chest radiography. Follow-up consisted of scheduled outpatient visits at 1 week and 1 month after discharge. Thereafter, annual follow-up was conducted either in person or by structured telephone interview to systematically assess adverse events and pneumothorax recurrence. The study’s final follow-up cutoff date is September 2025.

### Statistical approach

Data analysis was performed using SPSS 25.0 and R 4.3.0 (MatchIt package, version 4.5.0). To minimize baseline imbalances in prespecified potential confounders—including age, BMI, and comorbidities—between the two groups, we conducted 1:1 nearest-neighbor propensity score matching (PSM) without replacement. Although no statistically significant differences in baseline characteristics were observed between groups prior to matching, PSM was applied to reduce residual confounding and enhance covariate balance across all included variables, as confirmed by standardized mean differences (SMDs) < 0.1 for each covariate. The propensity score functions as a balancing score: conditional on its value, the distributions of observed baseline covariates are statistically comparable between the glucose protocol and parietal pleurectomy groups [23]. The propensity score was estimated using logistic regression, with group assignment (glucose protocol vs. parietal pleurectomy) as the outcome variable and the following prespecified baseline covariates as predictors: age, sex, BMI, smoking history (categorized as never, former, or current), pneumothorax laterality (left or right), diabetes mellitus, hypertension, and COPD diagnosis. The full propensity score logistic regression model, including regression coefficients and standard errors for all covariates, is provided in Supplementary Table S1. A caliper width of 0.2 standard deviations of the logit of the propensity score was applied, and matching was performed without replacement. Normally distributed continuous variables are presented as mean ± standard deviation (x̄ ± s); between-group comparisons after matching were conducted using the independent samples t-test. Categorical variables are presented as frequency (percentage) [n (%)]; group comparisons were performed using the χ² test or Fisher’s exact test, as appropriate. Baseline covariate balance after matching was assessed using standardized mean differences (SMDs), with an SMD < 0.1 interpreted as indicating adequate balance. Statistical significance was defined as *p* < 0.05. After matching, continuous variables were compared between groups using the independent-samples t-test, and categorical variables were compared using the chi-squared test, as commonly done in many published PS-matched studies. However, as noted in the Limitations section, these tests do not account for the matched nature of the data, and using paired tests or variance-adjusted methods would produce more precise estimates of treatment effects.

## Results

### PSM results

A total of 155 patients were enrolled (72 in the observation group and 83 in the control group). After 1:1 nearest-neighbor PSM with a caliper width of 0.2, 72 matched pairs (72 patients per group) were obtained, excluding 11 patients from the control group due to large differences in propensity scores (Table 2). As shown in Table 1, all baseline covariates (e.g., age, BMI, comorbidities) had standardized mean differences (SMDs) < 0.1 after matching (all P > 0.05), indicating that baseline characteristics were well balanced between the two groups. The p-value for BMI increased from 0.079 before matching to 0.862 after propensity score matching, indicating successful balancing of this covariate between groups. This improvement in statistical balance—alongside standardized mean differences (SMDs) < 0.1 for all matched variables—confirms that PSM effectively minimized baseline disparities in body composition. Perioperative variables included in the matched analysis were complete for the analytic cohort. No patients were lost to follow-up during the 1-year observation period. One-year recurrence status was ascertained for all 144 matched patients through scheduled outpatient visits whenever available, supported by chest radiography or CT when clinically indicated. For patients followed by structured telephone interview, suspected recurrence was counted only when supported by outside imaging reports, hospital records, or subsequent clinical documentation.

**Table 2:** Comparison of perioperative and outcome after PSM

outcome indicator	Observation Group (*n* = 72)	Control Group(*n* = 72)	t/x²/z	*P*
Surgical duration (min)	65.33 ± 2.43	84.92 ± 2.35	5.87	< 0.001
Intraoperative Blood Loss (mL)	50.00 ± 3.82	71.89 ± 4.62	3.69	< 0.001
Total Drainage Volume (mL)	551.39 ± 27.76	785.42 ± 57.83	3.52	< 0.001
Duration of Drainage Tube Indwelling (days)	4.42 ± 0.17	5.32 ± 0.21	3.45	0.001
Postoperative Length of Stay (days)	5.04 ± 0.16	6.31 ± 0.24	4.12	< 0.001
Postoperative Pain Score (VAS)				
Day 1	2.51 ± 0.08	3.10 ± 0.07	5.88	< 0.001
Day 2	1.92 ± 0.07	2.41 ± 0.07	5.29	< 0.001
Day 3	0.93 ± 0.07	1.28 ± 0.06	3.89	< 0.001

### Intraoperative air leakage

Intraoperative pulmonary air leakage following bullectomy occurred in 2 patients (2.8%) in the glucose protocol group and 1 patient (1.4%) in the parietal pleurectomy group, with no statistically significant between-group difference (*P* = 0.562; Fisher’s exact test). All air leaks were identified intraoperatively, localized to the staple line or resection margin, and successfully controlled using targeted interventions—including supplemental mechanical stapling or interrupted non-absorbable suturing—prior to chest closure and before initiation of pleurodesis. 

### Comparison of perioperative and outcome indicators between the two groups after PSM

#### Sensitivity analysis

To assess whether emphysema severity confounded the associations between pleurodesis strategy and perioperative outcomes, multivariable regression models were fitted with adjustment for Goddard emphysema severity category (mild, moderate, or severe). For continuous outcomes, linear regression was used, and treatment effects are reported as adjusted mean differences (glucose protocol minus parietal pleurectomy) with 95% confidence intervals (CI). After adjustment, the glucose protocol remained significantly associated with shorter operative duration (–19.6 min; 95% CI − 25.8 to − 13.4; *p* < 0.001), lower intraoperative blood loss (–21.9 mL; 95% CI − 31.9 to − 11.9; *p* < 0.001), lower total pleural drainage volume (–234.0 mL; 95% CI − 362.2 to − 105.8; *p* < 0.001), shorter chest tube dwell time (–0.90 days; 95% CI − 1.35 to − 0.45; *p* < 0.001), and shorter postoperative hospital stay (–1.27 days; 95% CI − 1.78 to − 0.76; *p* < 0.001). For binary outcomes, logistic regression models adjusted for emphysema severity showed no statistically significant associations with total complication rate (adjusted odds ratio [aOR] 1.22; 95% CI 0.55–2.71; *p* = 0.62) or 1‑year recurrence (aOR 1.42; 95% CI 0.42–4.84; *p* = 0.57). These findings confirm that the beneficial perioperative associations of the glucose protocol are independent of emphysema severity, while recurrence results remain underpowered. Detailed regression outputs are provided in Supplementary Table S2.

After propensity score matching, the glucose protocol group demonstrated significantly improved perioperative outcomes compared to the parietal pleurectomy group (all *P* < 0.05): shorter operative duration (65.33 ± 2.43 vs. 84.92 ± 2.35 min), reduced intraoperative blood loss (50.00 ± 3.82 vs. 71.89 ± 4.62 mL), lower total pleural drainage volume (551.39 ± 27.76 vs. 785.42 ± 57.83 mL), shorter chest tube indwelling time (4.42 ± 0.17 vs. 5.32 ± 0.21 days), and shorter postoperative length of hospital stay (5.04 ± 0.16 vs. 6.31 ± 0.24 days). Visual Analog Scale (VAS) pain scores on postoperative days 1–3 were also significantly lower in the glucose protocol group (all *P* < 0.001). However, no statistically significant differences were observed between the two groups in total complication rate (18.1% vs. 15.3%, *P* = 0.597) or 1-year pneumothorax recurrence rate (4.2% vs. 2.8%, *P* = 0.647) (Table 3).

**Table 3:** Detailed comparison of postoperative complications and recurrence rates after PSM

specific (%)	Observation group (*n* = 72)	Control group (*n* = 72)	t/x²/z	*P*
Persistent pulmonary air leak	3 (4.2%)	1 (1.4%)	-	0.346*
Pleural effusion	4 (5.6%)	3 (4.2%)	-	0.706*
Pulmonary infection	2 (2.8%)	3 (4.2%)	-	0.688*
Transient hyperglycemia	3 (4.2%)	0 (0.0%)	-	0.099*
Intercostal nerve injury	1 (1.4%)	3 (4.2%)	-	0.375*
Encapsulated pleural effusion	0 (0.0)	0 (0.0)	-	-
Incidence of Persistent Pleural Space	0 (0.0)	0 (0.0)	-	-
Total Complication Rate	18.1	15.3	χ²=0.28	0.597
1-Year Recurrence Rate	4.2	2.8	χ²=0.21	0.647

* indicates that the comparison was performed using Fisher’s exact test

## Discussion

The robustness of our findings is strengthened by propensity score matching (PSM), which generated well-balanced comparison groups and mitigated the selection bias inherent in this retrospective cohort study. Pleural fixation is a critical step in preventing pneumothorax recurrence after pulmonary bullae resection. For young, otherwise healthy patients, mechanical pleurodesis—such as parietal pleurectomy or pleuropexy—is widely regarded as the reference standard for achieving reliable and durable pleural symphysis [24]. However, this study focuses on a distinct and clinically challenging population: elderly patients with recurrent, diffuse, or multiple pulmonary bullae refractory to conservative management. In such patients, parietal pleurectomy is often technically demanding due to extensive surgical dissection, substantial intraoperative blood loss, severe postoperative pain, and pronounced impairment of baseline lung function [25]. Consequently, there is a compelling clinical need to identify an effective, minimally invasive alternative. Hypertonic glucose solution (50%) has been investigated for decades as a safe, inexpensive, and pro-inflammatory pleurodesis agent [26]. In this study, we compared the efficacy and safety of an enhanced glucose-based pleurodesis protocol—involving intraoperative hypertonic glucose spray followed by three-day postoperative intrapleural glucose perfusion—with parietal pleurectomy, using rigorous retrospective cohort methodology.

In this study, intraoperative hypertonic glucose (50%) spraying was selected as a representative chemical pleurodesis technique, and parietal pleurectomy was chosen as the reference mechanical pleurodesis procedure. A comparative analysis between these two approaches was conducted based on three complementary clinical and mechanistic criteria [10, 27, 28]: First, their mechanisms of action are fundamentally distinct. Parietal pleurectomy achieves anatomical obliteration of the pleural cavity by complete surgical stripping of the parietal pleura, thereby exposing the underlying endothoracic fascia and triggering a robust, fibroproliferative inflammatory response that yields strong, durable pleural symphysis. In contrast, intraoperative hypertonic glucose (50%) spraying induces pleurodesis primarily through hyperosmotic dehydration of mesothelial cells and localized chemical irritation, resulting in a milder, predominantly exudative-inflammatory reaction that promotes less robust but clinically sufficient adhesion formation—reflecting divergent pathophysiological pathways. Second, their clinical indications differ meaningfully. Parietal pleurectomy is particularly indicated for patients with recurrent spontaneous pneumothorax, extensive or bilateral pulmonary bullae, or concomitant pleural thickening; published evidence reports long-term recurrence rates below 2% in such cohorts. Third, their risk–benefit profiles are markedly dissimilar. While parietal pleurectomy offers near-permanent pleural closure, it entails substantial surgical trauma, requires advanced thoracic surgical expertise, and is associated with higher risks of intraoperative bleeding, prolonged postoperative pain, and postoperative pleural effusion. Conversely, intraoperative hypertonic glucose spraying is minimally invasive, causes significantly less tissue disruption, results in milder postoperative pain, and enables faster functional recovery—though it is associated with a higher long-term recurrence rate compared with pleurectomy.

This study introduces an enhanced pleurodesis protocol tailored for high-risk elderly patients, integrating intraoperative hypertonic glucose (50%) spray with postoperative fractionated intrapleural perfusion over three consecutive days. This multimodal strategy provides sustained chemical stimulation of the pleural surfaces, thereby promoting a coordinated inflammatory and fibroproliferative response that enhances the strength and durability of pleural symphysis—while minimizing surgical trauma and preserving baseline pulmonary function.

Compared with parietal pleurectomy, the glucose protocol demonstrated significantly improved perioperative outcomes—including shorter operative duration, reduced intraoperative blood loss, lower total pleural drainage volume, shorter chest tube indwelling time, shorter postoperative length of hospital stay, and lower Visual Analog Scale (VAS) pain scores on postoperative days 1–3 (all *P* < 0.05). Critically, no statistically significant differences were observed between the two groups in total complication rate or 1-year pneumothorax recurrence rate (both *P* > 0.05), confirming that the glucose protocol offers comparable safety and durability while providing substantial perioperative benefits for this high-risk elderly population.

The observed differences in operative duration and intraoperative blood loss are not unexpected, given the fundamentally distinct nature of the two procedures. Parietal pleurectomy necessitates extensive, layer-by-layer dissection of the parietal pleura from the underlying endothoracic fascia—a technically demanding maneuver that inevitably disrupts the dense capillary and lymphatic networks embedded within the chest wall, thereby contributing to greater blood loss and prolonged operative time [29, 30]. In contrast, intraoperative hypertonic glucose (50%) spraying involves only uniform topical application of the sclerosing agent onto the visceral and parietal pleural surfaces. This minimally invasive technique is rapid, technically straightforward, and avoids the mechanical trauma, tissue devascularization, and inflammatory cascade triggered by surgical stripping [31]. Nevertheless, these differences primarily reflect the inherent procedural invasiveness of the two interventions and should not be construed as a comprehensive assessment of total episode-related burden—which is more appropriately evaluated using objective, patient-centered postoperative recovery metrics, including length of hospital stay and chest tube dwell time. Critically, the glucose protocol conferred two clinically meaningful intraoperative advantages: significantly reduced anesthetic exposure time and a lower incidence of perioperative red blood cell transfusion—both of which are especially consequential for elderly patients with diminished physiologic reserve and heightened vulnerability to hemodynamic instability and anesthetic complications. Second, the higher total pleural drainage volume, longer chest tube indwelling time, and extended postoperative length of hospital stay observed in the parietal pleurectomy group are directly attributable to the extensive wound surface area and pronounced tissue fluid exudation induced by surgical stripping of the parietal pleura. In the glucose protocol group, a transient early increase in drainage volume may occur due to the hyperosmotic effect of intrapleural 50% glucose—causing acute mesothelial cell dehydration and localized exudative inflammation. However, because this approach avoids surgical incision, dissection, or tissue removal, exudate production declines rapidly after the initial inflammatory peak, consistent with the significantly shorter duration of chest tube drainage documented in this study [27]. Third, the markedly lower postoperative pain scores in the glucose protocol group reflect the substantially reduced tissue trauma associated with chemical versus mechanical pleurodesis. Parietal pleurectomy entails extensive dissection within the intercostal nerve–rich parietal pleural bed, inevitably provoking severe, prolonged neuropathic and nociceptive pain [32]. In contrast, chemical pleurodesis—exemplified by intraoperative hypertonic glucose (50%) spraying—induces pleural inflammation primarily through localized chemical irritation, resulting in generally milder and more readily controlled postoperative pain [33].

The present findings should be interpreted by separating two distinct dimensions: clinical effectiveness (prevention of recurrence and complications) and procedural invasiveness (surgical trauma and recovery burden). In this study, the glucose protocol and parietal pleurectomy showed comparable clinical effectiveness—no significant differences in 1-year recurrence (4.2% vs. 2.8%, *p* = 0.647) or total complications (18.1% vs. 15.3%, *p* = 0.597). In contrast, all invasiveness indicators (operative time, blood loss, drainage volume, chest tube duration, pain scores, and length of stay) significantly favored the glucose protocol (all *p* < 0.05), consistent with the less traumatic mechanism of chemical pleurodesis. This distinction has clinical relevance: for elderly high-risk patients with limited physiologic reserve, the glucose protocol’s reduced invasiveness may outweigh a numerically—though not statistically—higher recurrence risk, whereas parietal pleurectomy may be preferred when maximizing long-term recurrence prevention is paramount. Future prospective trials should evaluate these two dimensions as separate endpoints to better inform individualized treatment decisions.

A related and more fundamental question concerns the methodological validity of comparing a single-stage mechanical procedure with a multi-day chemical pleurodesis protocol. We acknowledge that these strategies differ fundamentally in the temporal distribution of therapeutic intervention: parietal pleurectomy delivers the entire intervention intraoperatively, whereas the glucose protocol distributes therapeutic exposure across a standardized 3-day postoperative window. Nevertheless, we contend—on clinical and methodological grounds—that this comparison is not only valid but essential, for three interrelated reasons.First, the 3-day instillation regimen was empirically grounded, not arbitrary. Multiple instillations are an established feature of hypertonic glucose pleurodesis in real-world practice. Fujino et al. reported a mean of 1.4 instillations (range 1–3) among successfully treated patients, with approximately 20% requiring two or three doses to achieve sustained air leak cessation [34]. The ongoing PLUG-II trial employs a protocol of glucose instillation on postoperative days 1 and 2 (with optional day 3 dosing based on air leak status). Similarly, Hashimoto et al. recently demonstrated successful pleurodesis following three sequential hypertonic glucose instillations in patients with refractory hepatic hydrothorax [35].

Mechanistically, durable pleural symphysis relies on sustained and tightly regulated pleural inflammation coupled with robust fibroblast activation. In contrast, single-dose intraoperative pleurodesis—such as the glucose spray technique described by Tsuboshima et al. in their 2018 cohort study of 376 patients with primary spontaneous pneumothorax—yielded a significant reduction in postoperative recurrence among young, low-risk individuals (hazard ratio, 0.15; 95% CI, 0.03–0.72; *p* = 0.014) [26].However, its efficacy in elderly patients with diffuse emphysematous bullae—characterized by impaired tissue repair, chronic inflammation, and extensive pleural surface involvement—is biologically uncertain. Our 3-day protocol therefore represents a rationally intensified strategy, calibrated to enhance the probability of complete and durable pleural symphysis in this higher-risk population. Second, the clinically meaningful metric for evaluating comparative burden is not daily procedural intensity but total episode-related resource utilization—most objectively captured by length of hospital stay. In our cohort, patients receiving the glucose protocol had a significantly shorter total hospital stay (5.04 vs. 6.31 days; *p* < 0.001), representing a mean reduction of 1.27 inpatient days per patient. This finding aligns with data from Jeong In Hong et al., who reported significantly shorter hospitalization with 50% glucose pleurodesis versus autologous blood patch (7.83 ± 4.75 vs. 9.11 ± 5.42 days) [36]. Critically, all glucose instillations were administered safely at the bedside via the existing indwelling chest tube, requiring only brief nursing time (estimated 5–10 min per dose×3 doses = 15–30 min total per patient), without additional invasive procedures, imaging guidance, or specialized equipment. Given the substantial cost and resource implications of each inpatient day, this net reduction in length of stay likely outweighs the minimal incremental nursing workload. Third, 50% hypertonic glucose is a well-established, low-cost pleurodesis agent with a favorable safety profile. As emphasized by the PLUG trial investigators, hyperosmolar glucose solution has been used for decades in several Asian countries owing to its “simplicity, safety, and low cost.” Although formal cost-effectiveness analysis was beyond the scope of this study, the convergence of three factors—shorter hospital stay, negligible drug acquisition cost (< USD $1 per dose), and minimal incremental personnel or infrastructure requirements—strongly suggests that the glucose protocol constitutes a resource-efficient alternative to parietal pleurectomy in elderly high-risk patients. Future prospective randomized trials should incorporate granular micro-costing, standardized assessment of procedural burden (e.g., nursing time, imaging use, analgesic requirements), and validated patient-reported outcome measures—including symptom burden and functional recovery—to rigorously quantify and compare the total episode burden of these two pleural symphysis strategies.

Notably, despite marked differences in procedural invasiveness and tissue trauma, no statistically significant difference was observed in 1-year pneumothorax recurrence rates between the glucose protocol and parietal pleurectomy groups. This absence of a statistically significant difference in long-term recurrence may be explained by three interrelated factors [37, 38]: First, contemporary video-assisted thoracoscopic surgery (VATS) bullectomy—performed in all patients—ensures precise resection of visible bullae and thorough inspection of the entire visceral pleura, thereby reducing the baseline risk of recurrence at its anatomical source and diminishing the relative contribution of the subsequent pleurodesis method to overall recurrence prevention. Second, unlike classic risk factors for primary spontaneous pneumothorax (e.g., young age, tall stature, low body mass index), our cohort comprised elderly patients with underlying emphysema. In this population, recurrence is predominantly driven by the diffuse, multifocal nature of emphysematous bullae rather than isolated apical blebs—rendering complete surgical eradication inherently challenging and elevating the importance of effective pleurodesis regardless of technique. Third—and most critically—the protocolized three-day postoperative intrapleural perfusion with 50% hypertonic glucose represents the pivotal innovation of this study. Unlike traditional single-dose intraoperative spraying—which is susceptible to suboptimal distribution due to gravitational effects, variable pleural adhesion geometry, and early drainage through chest tubes—this extended, fractionated delivery ensures sustained contact of the sclerosing agent with both visceral and parietal pleural surfaces, thereby enhancing the intensity and uniformity of the inflammatory–fibroproliferative response required for durable symphysis formation [27].

A critical question is whether the intraoperative spraying component of the glucose protocol is indispensable—or whether equivalent efficacy and safety could be achieved with postoperative intrapleural perfusion alone. Although this study was not powered or designed to resolve this mechanistic question, we posit that the intra- and postoperative components fulfill distinct yet synergistic roles. Intraoperative spraying, performed under direct thoracoscopic visualization, enables precise, uniform coverage of anatomically high-risk regions—namely the thoracic apex, costophrenic recesses, and paravertebral sulci—where gravitational dependency and preexisting pleural adhesions may impede homogeneous distribution of subsequently instilled solution. Postoperative fractionated perfusion, in turn, delivers sustained biochemical stimulation to drive a robust and durable inflammatory response, culminating in fibroproliferative pleural apposition. Whether a postoperative-only regimen would replicate these outcomes remains an important knowledge gap; de-escalation trials rigorously evaluating non-inferiority in both efficacy (e.g., recurrence-free survival) and safety (e.g., procedural complications, pain burden) are warranted. Pending such evidence, the integrated intra- and postoperative strategy described herein constitutes a clinically prudent, evidence-informed approach optimized to maximize the likelihood of complete and durable pleural symphysis.

Collectively, this pleurodesis protocol—integrating intraoperative targeted spraying with postoperative fractionated perfusion—delivers sustained, temporally staged stimulation of the parietal and visceral pleural surfaces, thereby promoting more robust, spatially homogeneous, and histologically durable pleural symphysis formation. As a result, no statistically significant difference was observed in 1-year pneumothorax recurrence–free survival between the two groups, although the study was not powered to establish non-inferiority. Parietal pleurectomy remains a mechanically intensive intervention associated with greater tissue disruption and prolonged recovery.

Additionally, a recognized concern with chemical pleurodesis is the potential for localized complications, including loculated pleural effusion or persistent residual pleural cavities, attributable to heterogeneous or incomplete pleural adhesion [11, 39]. To address this, we conducted a systematic, blinded review of postoperative chest imaging (CT or high-resolution radiography) in all enrolled patients. No cases of encapsulated pleural effusion or persistent residual pleural cavities were identified in either the glucose protocol or parietal pleurectomy group. This finding is clinically meaningful: it indicates that the enhanced three-day hypertonic glucose (50%) protocol—when integrated with procedural safeguards such as intraoperative uniform spray coverage, postoperative patient positioning to optimize solution distribution, and titrated low-volume perfusion doses—does not appear to increase the risk of adhesion-related structural complications beyond baseline levels. While these results support short-term safety, definitive validation of long-term pleural integrity and functional outcomes requires large-scale, prospective studies with standardized, longitudinal imaging surveillance.

All procedures were performed by attending thoracic surgeons within the same department using a standardized two-port VATS workflow; however, procedures were not necessarily performed by the same individual surgeon. Differences in surgeon experience, intraoperative judgment, and technical preference may have influenced both treatment selection and perioperative outcomes. Moreover, parietal pleurectomy is intrinsically more technically demanding than topical hypertonic glucose spraying because it requires pleural incision, dissection, and hemostasis over a broad parietal pleural surface. A recent 2025 meta-analysis of 15 studies involving 2732 patients confirmed that parietal pleurectomy is independently associated with significantly longer operative time (MD = 15.87 min, 95% CI 11.30–20.44) and greater intraoperative blood loss (MD = 14.62 mL, 95% CI 8.58–20.66) compared even with pleural abrasion, a less extensive mechanical pleurodesis procedure [40]. Taken together, these findings indicate that the observed differences in operative time and blood loss largely reflect inherent differences in the technical complexity and procedural invasiveness of the two interventions, rather than a treatment effect attributable solely to the pleurodesis agent itself. This consideration further supports interpreting operative time and blood loss as measures of procedural burden rather than as direct measures of recurrence-prevention effectiveness. Future studies employing standardized operative protocols and controlling for surgeon-specific effects are warranted to more precisely quantify the relative contributions of technical complexity versus pleurodesis strategy to the observed perioperative differences.

In this study, the 1-year pneumothorax recurrence rate in the glucose protocol group was numerically higher than that in the parietal pleurectomy group (4.2% vs. 2.8%); however, this difference did not reach statistical significance (*P* = 0.647). This null finding is likely attributable to limited statistical power for the recurrence endpoint, given the relatively small sample size—particularly after propensity score matching, which further reduced the effective cohort size. Indeed, post hoc power analysis indicated that our study had only 22% power to detect a difference of this magnitude (assuming α = 0.05, two-sided test), well below the conventional 80% threshold. In contrast, Tsuboshima et al. [26], who reported a statistically significant reduction in recurrence with glucose pleurodesis, enrolled a substantially larger cohort (*n* = 376) and achieved 92% power to detect a similar effect size. Thus, while no statistically significant difference was detected in long-term recurrence rates, the study was underpowered for this endpoint and did not prespecify a non-inferiority margin. Definitive confirmation requires adequately powered, prospective studies with systematic, long-term imaging and clinical follow-up.

Several additional limitations should be emphasized. First, treatment allocation was not randomized and was influenced by attending surgeon preference, anatomical considerations, anticipated tolerance of pleurectomy, comorbidity burden, patient/family preference, and evolving departmental practice over time. Although PSM improved balance for measured baseline covariates, unmeasured factors such as frailty, ASA class, pulmonary function, pleural adhesions, surgeon experience, and time-period effects may have introduced residual confounding. Second, the term “high-risk” was used descriptively rather than according to a validated perioperative risk score because complete ASA class, frailty indices, and pulmonary function data were not uniformly available. Third, we did not account for the matched-pairs nature of the data when conducting statistical tests in the propensity-score matched sample. Several studies have shown that using independent-sample tests post-PSM can lead to inflated type I error rates and understated standard errors, particularly when the treatment-selection process is strong [41]. Instead, tests that account for the matched nature of the data, such as the paired t-test or McNemar’s test, are recommended for matched designs and have been shown to produce more conservative, theoretically sound estimates. Fourth, VAS pain assessment was not blinded, and subjective pain outcomes may be affected by performance and detection bias, although both groups received the same institutional analgesic protocol. Fifth, the study was underpowered for 1-year recurrence, and absence of a statistically significant difference does not demonstrate equivalence or non-inferiority. Sixth, while parietal pleurectomy and intraoperative glucose spraying are inherently different in technical complexity, the present study did not account for potential confounding effects of surgeon-specific operative styles and individual procedural variations, such as the extent of pleural dissection and the volume of glucose solution used for spraying. These unmeasured factors may have contributed to residual confounding of the observed between-group differences in operative time and blood loss. Seventh, all procedures were performed within a single department, which may limit generalizability. Furthermore, although all enrolled patients exhibited CT-confirmed diffuse emphysema, only a subset met formal spirometric criteria for chronic obstructive pulmonary disease (COPD), highlighting the heterogeneity of underlying airway pathology within this high-risk population.

This discrepancy reflects a well-documented clinical–epidemiological gap: a substantial proportion of individuals with CT-confirmed emphysema—particularly those with mild-to-moderate parenchymal destruction—lack a formal COPD diagnosis, commonly due to absence of prior spirometry or failure to meet the post-bronchodilator FEV1/FVC < 0.7 threshold [42]. Large-scale epidemiological studies indicate that over 90% of individuals with spirometrically confirmed COPD are classified as GOLD stages I–II and remain undiagnosed in routine clinical practice [42]. Similarly, the COPDGene study demonstrated that more than half of current or former smokers with preserved spirometry (FEV1/FVC ≥ 0.7 and FEV1 ≥ 80% predicted) exhibited significant respiratory impairment—including CT-documented emphysema, gas trapping, and airway wall thickening—that was not captured by conventional spirometry-based COPD definitions [43]. Consequently, using documented COPD diagnosis as a surrogate for underlying chronic airway and parenchymal disease likely underestimates the true burden of structural and functional impairment in this cohort—a recognized limitation of retrospective studies reliant on administrative or clinic-based diagnostic coding.

## Conclusions

In this retrospective, propensity-score matched cohort study involving elderly patients at high surgical risk with recurrent, diffuse pulmonary bullae, an enhanced pleurodesis protocol—comprising intraoperative thoracoscopic instillation of 50% hypertonic glucose solution followed by three daily doses of fractionated intrapleural glucose perfusion—was associated with significantly shorter operative duration, reduced intraoperative blood loss, lower cumulative pleural drainage volume, shorter chest tube dwell time, reduced postoperative length of stay, and lower early postoperative pain scores compared with parietal pleurectomy. No statistically significant differences were observed in overall complication rates or 1-year pneumothorax recurrence–free survival. These findings indicate that the glucose-based protocol may serve as a less invasive alternative to mechanical pleurectomy in this vulnerable population, providing comparable short-term safety in preventing recurrent pneumothorax. However, the study was not designed to determine the optimal overall treatment strategy for secondary spontaneous pneumothorax, nor does it permit quantification of the incremental benefit conferred by pleurodesis beyond bullectomy alone. Given its retrospective, single-center design and limited statistical power for the primary efficacy endpoint (1-year recurrence–free survival), these results should be interpreted with caution and are appropriately characterized as hypothesis-generating rather than confirmatory. Definitive validation requires large-scale, multicenter, prospective randomized controlled trials with prespecified non-inferiority margins, extended follow-up (≥ 2 years), and assessment of real-world implementation feasibility to rigorously evaluate comparative clinical effectiveness, long-term durability of pleurodesis, and generalizability across diverse high-risk cohorts.

## Supplementary Information

Supplementary Material 1.

Supplementary Material 2.

## Data Availability

The datasets generated during and analyzed during the current study are not publicly available due to privacy and legal restrictions but are available from the corresponding author on reasonable request.

## Abbreviations

BMI: Body Mass Index
COPD: Chronic Obstructive Pulmonary Disease
PSM: Propensity Score Matching
SMD: Standardized Mean Difference
VAS: Visual Analogue Scale
