# Supplementary material for: Modified enhanced pleural fixation: combined intra- and postoperative hypertonic glucose in elderly and high-risk pulmonary bullae patients — a retrospective cohort study
Source: BMC Surg. 2026 May 21;26:538. doi: 10.1186/s12893-026-03826-y (PMC13471438; doi:10.1186/s12893-026-03826-y)
Supplement: Supplementary file 2 [file 12893_2026_3826_MOESM2_ESM.pdf]

1. Supplementary Table S1

Propensity score logistic regression model for treatment allocation (glucose protocol vs. parietal pleurectomy).

Variable	Coefficient (β)	Standard Error (SE)	z	p
Intercept	−3.358	1.229	−2.733	0.006
Age (years)	−0.027	0.018	−1.496	0.135
Sex (Male vs. Female)	−0.053	0.270	−0.197	0.844
BMI (kg/m²)	−0.041	0.053	−0.780	0.435
Smoking history (Yes vs. No)	−0.146	0.219	−0.669	0.503
Pneumothorax laterality (Right vs. Left)	−0.126	0.192	−0.658	0.511
Diabetes (Yes vs. No)	0.120	0.311	0.387	0.699
Hypertension (Yes vs. No)	−0.209	0.295	−0.710	0.478
COPD (Yes vs. No)	−0.058	0.346	−0.168	0.867

Note: The dependent variable was treatment group (glucose protocol = 1, parietal pleurectomy = 0). The model was estimated using data from all 155 patients before propensity score matching. Goddard emphysema severity was subsequently used as an adjustment covariate in sensitivity analyses (Supplementary Table S2) rather than in the propensity score model.

2. Supplementary Table S2

Adjusted treatment effects from multivariable regression models controlling for Goddard emphysema severity category (mild, moderate, severe).

Outcome	Model type	Adjusted effect (glucose vs. pleurectomy)	95% CI	p
Operative duration (min)	Linear regression	Mean difference –19.6	–25.8 to –13.4	<0.001
Intraoperative blood loss (mL)		Mean difference –21.9	–31.9 to –11.9	<0.001
Total pleural drainage volume (mL)		Mean difference –234.0	–362.2 to –105.8	<0.001
Chest tube dwell time (days)		Mean difference –0.90	–1.35 to –0.45	<0.001
Postoperative length of stay (days)		Mean difference –1.27	–1.78 to –0.76	<0.001

Total complication rate	Logistic regression	Adjusted OR 1.22	0.55 to 2.71	0.62
1-year pneumothorax recurrence		Adjusted OR 1.42	0.42 to 4.84	0.57

Supplementary Table S2 *Adjusted treatment effects from multivariable regression models controlling for Goddard emphysema severity category (mild, moderate, severe).*

All models included treatment group (glucose=1, pleurectomy=0) and Goddard severity (indicator variables for moderate and severe vs. mild).

3. Supplementary Figure S1

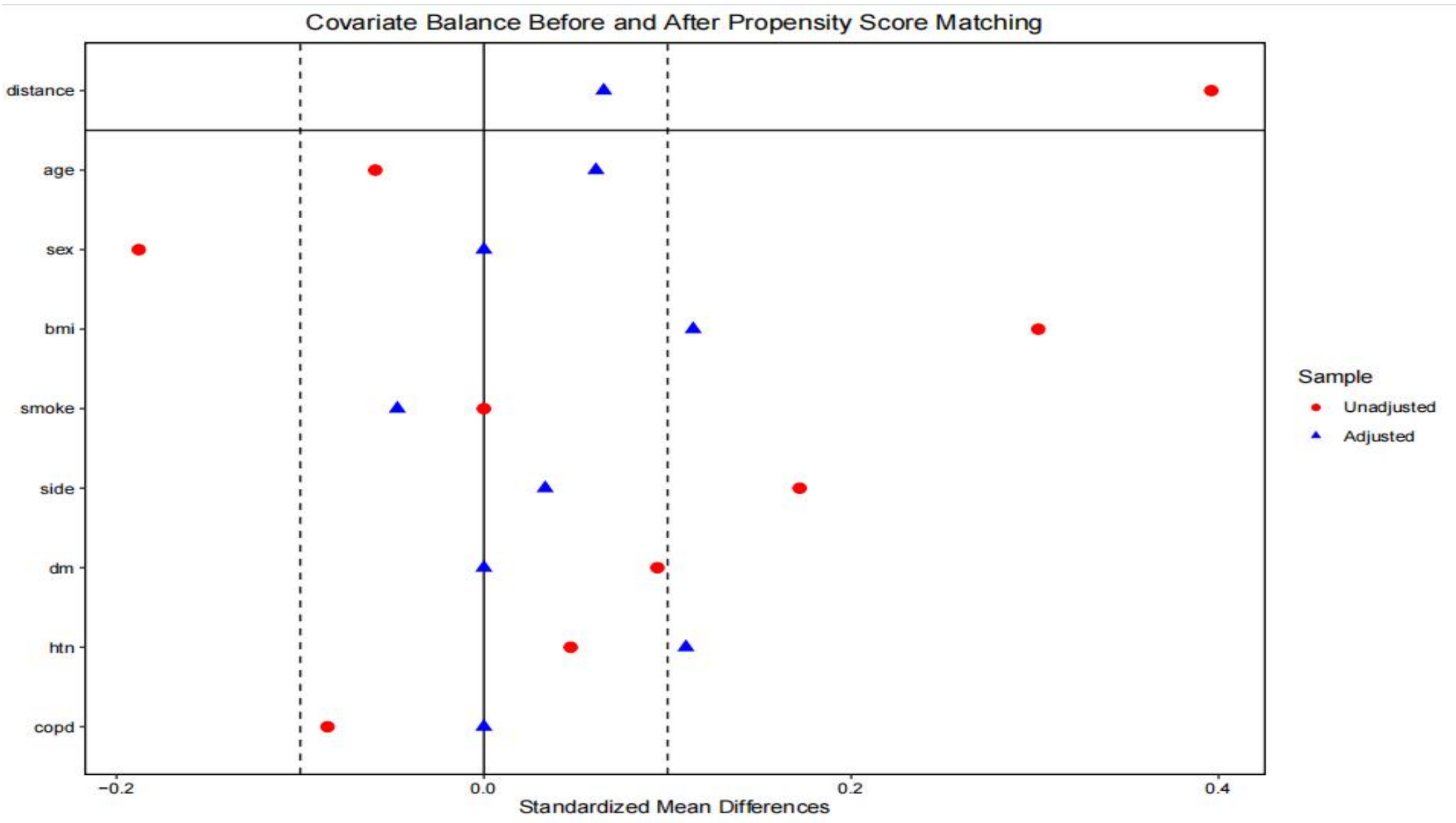

Love plot of covariate balance before and after propensity score matching.

Standardized mean differences (SMDs) for each covariate are shown before matching (red circles) and after matching (blue triangles). The dashed vertical line indicates the SMD threshold of 0.1, below which covariate balance is considered adequate. All covariates achieved SMDs < 0.1 after matching, confirming that the propensity score model effectively balanced baseline characteristics between groups.
